# A stereo matching algorithm based on the improved PSMNet

**DOI:** 10.1371/journal.pone.0251657

**Published:** 2021-08-19

**Authors:** Zedong Huang, Jinan Gu, Jing Li, Xuefei Yu

**Affiliations:** School of Mechanical Engineering, Jiangsu University, Zhenjiang, China; Taipei Medical University, TAIWAN

## Abstract

Deep learning based on a convolutional neural network (CNN) has been successfully applied to stereo matching. Compared with the traditional method, the speed and accuracy of this method have been greatly improved. However, the existing stereo matching framework based on a CNN often encounters two problems. First, the existing stereo matching network has many parameters, which leads to the matching running time being too long. Second, the disparity estimation is inadequate in some regions where reflections, repeated textures, and fine structures may lead to ill-posed problems. Through the lightweight improvement of the PSMNet (Pyramid Stereo Matching Network) model, the common matching effect of ill-conditioned areas such as repeated texture areas and weak texture areas is solved. In the feature extraction part, ResNeXt is introduced to learn unitary feature extraction, and the ASPP (Atrous Spatial Pyramid Pooling) module is trained to extract multiscale spatial feature information. The feature fusion module is designed to effectively fuse the feature information of different scales to construct the matching cost volume. The improved 3D CNN uses the stacked encoding and decoding structure to further regularize the matching cost volume and obtain the corresponding relationship between feature points under different parallax conditions. Finally, the disparity map is obtained by a regression. We evaluate our method on the Scene Flow, KITTI 2012, and KITTI 2015 stereo datasets. The experiments show that the proposed stereo matching network achieves a comparable prediction accuracy and much faster running speed compared with PSMNet.

## Introduction

**Fig 1:**
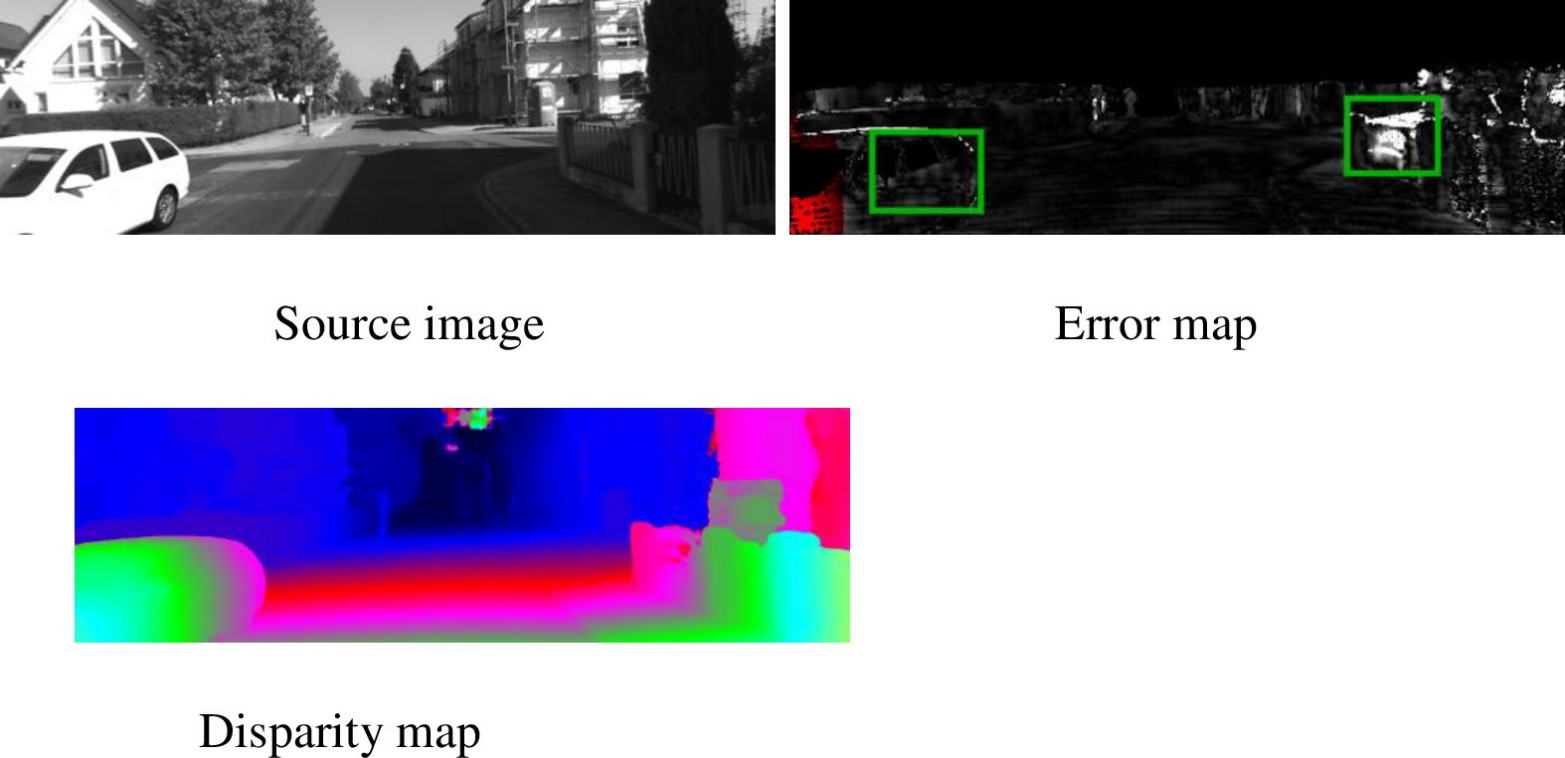
The disparity estimation results of the PSMNet algorithm in the ill-conditioned region. The error map scales linearly between 0 (black) and 5 (white) pixel errors. The green box in the figure shows a large error in the disparity estimation of the vehicle and wall reflection areas.

Stereo matching is the process of calculating the corresponding point deviation from stereo color image pairs to obtain a dense disparity map. It is widely used in automatic driving, 3D reconstruction, robot navigation, and other fields. As the stereo vision system’s core technology, stereo matching accuracy determines the performance of the entire system. Due to the presence of noise, repeated textures, low textures, occlusion, and other ill-conditioned areas and the lighting conditions, how to obtain an accurate disparity map efficiently and quickly is still a considerable challenge. As shown in the green box in Fig 1, the matching effect of repeated texture regions such as windows and roads is low. The traditional research focuses on loss calculation and disparity optimization. First, an excellent metric function has been designed to calculate the matching loss [1,2]. Second, local or global methods have been used to allocate disparity values [3,4] for each pixel. These algorithms all use shallow artificial functions and cannot obtain the correct results for ill-conditioned regions.

In recent years, the convolutional neural network (CNN) has made significant breakthroughs in many visual tasks such as object detection [5–7], semantic segmentation [8–10], etc.; and the application of deep learning in stereo matching is also ongoing. Convolution neural networks can extract robust features from images, which is suitable for learning the similarity between image blocks. To further improve a model’s global optimization ability, the end-to-end stereo matching method [11,12] integrates the entire process of disparity prediction into the CNN model of a region with fuzzy matching. However, this algorithm mostly uses a one-dimensional correlation algorithm along the parallax direction, which loses disparity dimension characteristics. The 3D convolution neural network (3DCNN) was introduced to stereo matching to make the model understand the global semantic information in three sizes to better understand a scene’s contextual information. The 3DCNN has been introduced into a stereo matching algorithm, which positively affects the modeling of the matching process. Nevertheless, it also increases the memory costs and the amount of computation by tens of times. To make full use of the global environment information, the pyramid pool structure is introduced into stereo matching. The matching cost volume is constructed by aggregating the environmental information of different scales and different positions. However, compared with the Atrous Spatial Pyramid Pooling (ASPP) module in DeepLab, spatial pyramid pooling has less information computation and a smaller receptive field range when it can also obtain multiscale information.

All of these methods reflect the application value of this study. This paper thoroughly studies the extraction method of multiscale spatial feature information, the stereo matching robustness of ill-formed regions, etc. We propose an improved Pyramid Stereo Matching Network (PSMNet) [13] algorithm to estimate various scenes’ disparities. The developed method has been painstakingly tested and compared with the usual methods. The remainder of this article is organized as follows. Section 2 describes the related works, and Section 3 introduces our proposed method, including the vast context learning network and stacked hourglass (encoder-decoder) architecture. In Section 4, we present the extensive experiments with an ablation study that were conducted and compare the accuracy results with those of the benchmarks. Section 5 summarizes this research and future efforts.

The stereo matching algorithm based on the convolution neural network can be divided into two categories [14]: deep learning methods combined with traditional methods and end-to-end deep learning stereo matching algorithms. The combination of deep learning and a conventional algorithm applies a deep learning algorithm to the steps of a conventional matching algorithm to learn the matching costs, cost aggregation, etc., and reduce the error caused by human design. Based on the end-to-end method, the left and right images are directly input, and the disparity image is output. The deep learning method is used to learn mapping from the original data to the desired outcome.

Regarding a stereo matching algorithm combining deep learning with a traditional algorithm, for the first time, Jure Zbontar and LeCun [15] take paired small image blocks and ground truths as the input and the matching costs as the output and train a matching cost CNN with a supervised learning network (MC-CNN). Then, they use the cross-cost aggregation method to aggregate the matching costs, use the SGM method to realize the smoothness constraint, check the left and the right consistency of the occluded area, and obtain a final disparity map through the median filter and bilateral filter. Williem et al. [16] proposed a self-guided cost aggregation method based on the deep convolution network and applied it to local stereo matching. The deep learning network of this method is composed of two subnetworks: emotional weight and descent filtering. In the system, the feature reconstruction loss and pixel mean square loss function are combined to maintain the edge characteristics; however, combining deep learning with traditional algorithms has considerable limitations. First, to calculate the matching costs under different disparities, the network contains multiple forward channels, which will cause a tremendous computational burden. Second, the pixels in the occluded area cannot be used for training, so the network cannot effectively estimate the disparity in the occluded area. Third, the system is not an end-to-end network and cannot directly generate the disparity map, which needs postprocessing. Because many network parameters are selected based on experience, the performance and generalization ability of the network are greatly restricted.

To solve the problems of image processing technology mentioned above, deep end-to-end learning has been used. Mayer et al. [11] first applied the end-to-end neural network model to the stereo matching field in 2016, established a large-scale synthetic data set, and trained the end-to-end CNN DispNet. The algorithm uses the automatic coding-decoding structure to directly output the disparity map without postprocessing steps and with high accuracy. Kang et al. [17] studied the algorithm that has a harmful matching effect in depth discontinuity and low texture areas. The improved learning algorithm of the DispNet neural network was proposed. The algorithm uses the extended convolution to build the feature extraction module of the context pyramid, builds the matching generation calculation method based on an image block, and introduces the disparity gradient information into the loss function for postprocessing to maintain the local details. Pang et al. proposed a cascaded residual learning (CRL) [12] network based on DispNet. The network is divided into two parts: DispFullNet and DispResNet. The former outputs the initial disparity map. The latter optimizes the initial disparity map by calculating the multiscale residuals. The two parts of the network are finally combined to form the final disparity map. Kendall et al. [18] proposed a GC-Net structure with an excellent effect. The system uses 3D convolution to obtain more contextual information and realize disparity estimation at the subpixel level. To obtain more essential information from images, some scholars use a convolutional neural network with depth feature fusion and multiscale image information extraction. Chang and Chen [13] proposed the PSMNet structure based on image semantic segmentation and global environment information. The network consists of two modules: pyramid pooling and a 3D convolution neural network. The pyramid pooling module builds the matching cost volume by aggregating the environmental information of different scales and locations through spatial pyramid pooling (SPP) [19] and nested convolution [20]. The 3D convolutional neural network module adjusts the matching cost volume by combining multiple stacked hourglass networks with intermediate supervision and improves global information utilization.

Compared with traditional methods, PSMNet has dramatically improved stereo matching accuracy. However, there are still some technical difficulties in estimating disparities for PSMNet in ill-posed regions, such as reflective surfaces, repetitive patterns, thin structures, and textureless areas. Therefore, it is necessary to combine the contextual information and increase the range of the receptive field. However, for the ordinary convolution layer, the size of the receptive field is directly related to the size of the convolution kernel. If a larger receptive field is needed, a larger convolution kernel can be selected. The other method is to cascade multiple convolution layers. These methods will increase the number of parameters and the computational costs, which is contrary to the original intention of this study. However, although PSMNet significantly improves stereo matching accuracy, it dramatically increases the network’s depth, followed by a rapid increase in the number of network parameters, and the calculation costs are also increasingly higher.

Focusing on the above problems, we propose an end-to-end neural network model. The model takes the existing excellent stereo matching end-to-end network PSMNet as the benchmark and improves its core feature extraction module and three-dimensional convolution module. The atrous convolution can expand the range of the receptive field without increasing the number of parameters. Even by adjusting the expansion rate, convolution layers with different receptive field sizes can be obtained. The void part will lose information. To solve this problem, the ASPP structure is introduced in this paper. By adjusting the ResNeXt [21] system and using ResNeXt + ASPP instead of ResNet + SPP to maintain accuracy, the receptive field can be enlarged, and spatial information at different scales can be obtained. In addition, through a feature fusion module, the feature information of different scales is fused. The 3D stacked hourglass aggregation network proposed in PSMNet is modified to further improve performance and decrease inference computational costs. Furthermore, 1×1×1 3D convolution is employed in the shortcut connections within each hourglass module without increasing computational costs.

## Materials & methods

The structure of the proposed improved PSMNet is shown in Fig 3. The structure consists of three parts: feature extraction, 3D convolution and the disparity regression. The detailed structures of the improved PSMNet are listed in Table 1, where B is the atrous convolution expansion rate, BN is batch regularization, ReLU denotes using the ReLU activation function, h is the height of the image, W is the image width, D is the maximum disparity value, concat is the cascading operation of the feature map, SElayer is the weight assigned to each feature map, and deconv is the deconvolution layer.

**Table 1:** Parameters of the improved PSMNet architecture.

Name	Layer setting	Output dimension
input		*H*×*W*×3
Feature Extraction (ResNeXt Module)		
Conv0_1	3 × 3, 32	$\frac{1}{2}H\times \frac{1}{2}W\times 32$
Conv0_2	3 × 3, 32	$\frac{1}{2}H\times \frac{1}{2}W\times 32$
Conv0_3	3 × 3, 32	$\frac{1}{2}H\times \frac{1}{2}W\times 32$
Conv1_x	$\left[\begin{array}{c} 3\times 3,32\\ 3\times 3,32 \end{array},C=32\right]\times 3$	$\frac{1}{2}H\times \frac{1}{2}W\times 32$
Conv2_x	$\left[\begin{array}{c} 3\times 3,64\\ 3\times 3,64 \end{array},C=32\right]\times 16$	$\frac{1}{4}H\times \frac{1}{4}W\times 64$
Conv3_x	$\left[\frac{3\times 3,128}{3\times 3,128},C=32\right]\times 3$	$\frac{1}{4}H\times \frac{1}{4}W\times 128$
Conv4_x	$\left[\frac{3\times 3,128}{3\times 3,128},C=32\right]\times 3,dila=2$	$\frac{1}{4}H\times \frac{1}{4}W\times 128$
Feature Extraction (ASPP Module)		
branch_1	3 × 3, 32, *B* = 6 1×1, 32 *bilinear*	$\frac{1}{4}H\times \frac{1}{4}W\times 32$
branch_2	3 × 3, 32, *B* = 12 1×1, 32 *bilinear*	$\frac{1}{4}H\times \frac{1}{4}W\times 32$
branch_3	3 × 3, 32, *B* = 18 1×1, 32 *bilinear*	$\frac{1}{4}H\times \frac{1}{4}W\times 32$
branch_4	3 × 3, 32, *B* = 24 1×1, 32 *bilinear*	$\frac{1}{4}H\times \frac{1}{4}W\times 32$
Feature Extraction (Fusion Module)		
Concat	Concat [branch_4, branch_3, branch_2, branch_1]	$\frac{1}{4}H\times \frac{1}{4}W\times 128$
SE	SElayer	$\frac{1}{4}H\times \frac{1}{4}W\times 128$
Conv5	3 × 3, 32	$\frac{1}{4}H\times \frac{1}{4}W\times 32$
Conv6	3 × 3, 128	$\frac{1}{4}H\times \frac{1}{4}W\times 128$
	Concat [Conv4_x,Conv5]	
Conv	1× 1, 32	$\frac{1}{4}H\times \frac{1}{4}W\times 32$
3D CNN (Cost volume)		
Concat left-shifted right		$\frac{1}{4}D\times \frac{1}{4}H\times \frac{1}{4}W\times 64$
3D CNN (stacked hourglass)		
3DConv0	3×3×3,32	$\frac{1}{4}D\times \frac{1}{4}H\times \frac{1}{4}W\times 32$
	3×3×3,32	
3DConv1	3×3×3,32	$\frac{1}{4}D\times \frac{1}{4}H\times \frac{1}{4}W\times 32$
	3×3×3,32	
	Add 3DConv0	
3Dstack1_1	3×3×3,64	$\frac{1}{8}D\times \frac{1}{8}H\times \frac{1}{8}W\times 64$
	3×3×3,64	
3Dstack1_2	3×3×3,64	$\frac{1}{16}D\times \frac{1}{16}H\times \frac{1}{16}W\times 64$
	3×3×3,64	
3Dstack1_3	deconv3×3×3,64	$\frac{1}{8}D\times \frac{1}{8}H\times \frac{1}{8}W\times 64$
	Add 3Dstack1_1	
3Dstack1_4	deconv3×3×3,32	$\frac{1}{4}D\times \frac{1}{4}H\times \frac{1}{4}W\times 32$
	Add 3DConv1	
3Dstack2_1	3×3×3,64	$\frac{1}{8}D\times \frac{1}{8}H\times \frac{1}{8}W\times 64$
	3×3×3,64	
3Dstack2_2	3×3×3,128	$\frac{1}{16}D\times \frac{1}{16}H\times \frac{1}{16}W\times 64$
	3×3×3,128	
3Dstack2_3	deconv3×3×3,64	$\frac{1}{8}D\times \frac{1}{8}H\times \frac{1}{8}W\times 64$
	Add 3Dstack2_1	
3Dstack2_4	deconv3×3×3,32	$\frac{1}{4}D\times \frac{1}{4}H\times \frac{1}{4}W\times 32$
	Add 3Dstack1_4	
3Dstack3_1	3×3×3,64	$\frac{1}{8}D\times \frac{1}{8}H\times \frac{1}{8}W\times 64$
	3×3×3,64	
3Dstack3_2	3×3×3,128	$\frac{1}{16}D\times \frac{1}{16}H\times \frac{1}{16}W\times 64$
	3×3×3,128	
3Dstack3_3	deconv3×3×3,64	$\frac{1}{8}D\times \frac{1}{8}H\times \frac{1}{8}W\times 64$
	Add 3Dstack3_1	
3Dstack3_4	deconv3×3×3,32	$\frac{1}{4}D\times \frac{1}{4}H\times \frac{1}{4}W\times 32$
	Add 3Dstack2_4	
Output_0	3×3×3,32, input = 3DConv0	$\frac{1}{4}D\times \frac{1}{4}H\times \frac{1}{4}W\times 1$
	3×3×3,1	
Output_1	3×3×3,32, input = 3Dstack1_4	$\frac{1}{4}D\times \frac{1}{4}H\times \frac{1}{4}W\times 1$
	3×3×3,1	
Output_2	3×3×3,32, input = 3Dstack2_4	$\frac{1}{4}D\times \frac{1}{4}H\times \frac{1}{4}W\times 1$
	3×3×3,1	
Output_3	3×3×3,32, input = 3Dstack3_4	$\frac{1}{4}D\times \frac{1}{4}H\times \frac{1}{4}W\times 1$
	3×3×3,1	
4 Output [Output_0, Output_1, Output_2, Output_3]		
Upsampling	Bilinear interpolation	*D*×*H*×*W*
	Disparity regression	*H*×*W*

**Fig 2:**
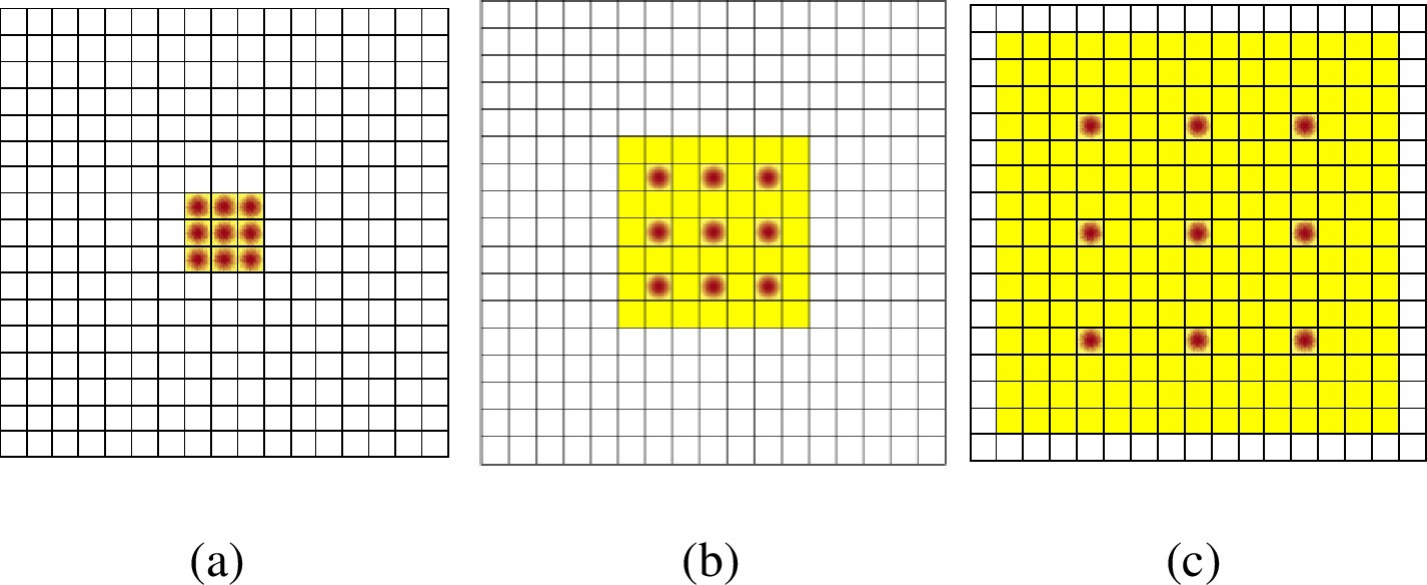
Schematic diagram of the atrous convolution.

**Fig 3:**
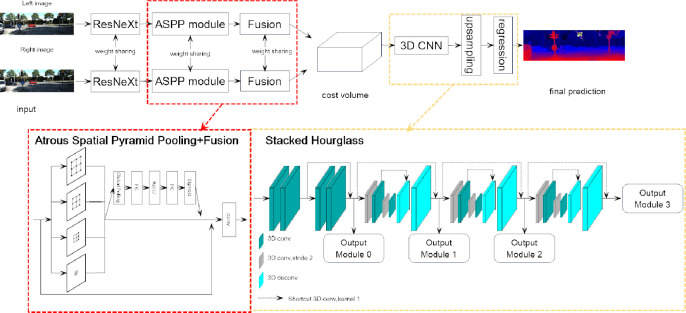
The pipeline of the proposed improved PSMNet network. The left and right images are input into two weighted residual convolution neural networks used to extract features, and the ASPP module is used to obtain the contextual information of images. Then, the left and right image features are connected to form a 4-D cost space, and the costs are regularized by a multiscale 3D CNN network. Finally, an accurate disparity map is obtained by disparity regression.

For feature extraction, due to the reflection and occlusion problems in real-life scenes, the network needs to capture contextual information on a larger scale on multiple scales. Therefore, inspired by DeepLabV2 [22], we adopt the ASPP structure. According to reference [21], compared with ResNet, ResNeXt improves accuracy without increasing the complexity of the parameters and reduces the number of superparameters. Therefore, this paper introduces ResNeXt to the feature extraction module. Based on the ResNeXt structure, three cascaded kernels are used as the 3×3 convolution filter in the first convolutional layer to reduce the number of parameters while obtaining the same perceptual domain. As shown in Table 1, the output image size is $\frac{1}{4}H\times \frac{1}{4}W$. In addition, conv2_x, conv3_x, and conv4_x are the bottleneck models of the residual networks. For ASPP, four parallel branches are formed by the atrous convolution with different expansion rates. The concept of the dilation rate is introduced in the atrous convolution. Here, Fig 2A represents the atrous convolution with a 3×3 convolution kernel and a dilation rate of one, that is, the ordinary convolution; Fig 2B shows the atrous convolution with a 3×3 convolution kernel and an expansion rate of 2. Since only 9 points and 3×3 convolution kernels are involved, other issues are not addressed. Therefore, even if the convolution kernel’s size is only as shown in Fig 2C, the receptive field of the cavity with a 3×3 convolution kernel and an expansion rate of 4 has increased to 15×15. The atrous convolution, which increases the receptive field without changing the image resolution, can completely replace the pooling operation. The output of each convolution contains a broad range of image feature information. The feature map extracts the spatial information of different scales through four branches. The four units can complement each other, and the output is accumulated to obtain the feature map with different scales and a broad range of receptive fields. The model structure is shown in Fig 3.

Because the importance of the information contained in each feature graph is different, inspired by SENet [23], we give each feature graph a specific weight, and the calculation method of the weight is shown in Fig 1. The feature map group is transformed into a one-dimensional feature vector by global average pooling. The bottleneck structure is used to limit the number of parameters, and then the weight of each channel is obtained by using the sigmoid function. The bottleneck structure is composed of two 1×1 convolution layers and one ReLU activation layer. The first convolution layer compresses the number of channels to one-sixteenth of the original number. After the ReLU function is activated, the number of channels is restored by the second convolution layer, and the weighted coefficients are multiplied by the corresponding feature map group to obtain the weighted feature map group. Then, the initial feature map is cascaded with the weighted feature map group through the jump connection, and the number of channels is compressed to 32 through the convolution layer to obtain the final fused feature map group. Two feature extraction networks with the same structure and shared weight are used to extract the feature information from the input left and right images to ensure that the feature values of the matching points on the corresponding channel are the same.

For the 3D convolution, we connect the left feature map and the corresponding right feature map under each parallax to obtain a 4D volume, which contains four dimensions: the height, width, parallax and feature. This method preserves the feature dimension and integrates it into the matching cost volume. Compared with other distance metric functions, this method can effectively improve the performance of the stereo matching network. After adding the disparity dimension, the dimension of the 4D cost volume is $64\times \frac{1}{4}D\times \frac{1}{4}H\times \frac{1}{4}W$.

Given the obtained matching cost volume, we need to learn a model to aggregate and regularize the disparity information and environmental feature information. Similar to PSMNet, we adopt the 3D CNN method, which can learn feature representations using the height, width, and disparity. The problem of too high computational costs when using the 3D CNN is solved by using encoding and decoding structures. The method consists of a pre-hourglass module and three stacked 3D hourglass networks to regularize the feature volumes. As shown in Fig 3, the pre-hourglass module consists of four 3D convolutions with batch normalization and ReLU. Three stacked 3D hourglass networks used to refine low-texture ambiguities and occlusion parts using encoder-decoder structures follow. Compared with the 3D aggregation network of PSMNet, we have several vital modifications to improve the performance and increase the inference speed, and the details of the structure are shown in Fig 3 and Table 1. The pre-hourglass module and three stacked 3D hourglass networks are connected to output modules. Each output module predicts a disparity map.

For the pre-hourglass module, we add one more auxiliary output module (output module 0, see Fig 1) for the pre-hourglass module features. The extra additional loss makes the network learn better features at lower layers, which also benefits the final prediction. Then, the residual connections between different output modules are removed. Thus, auxiliary output modules (output modules 0, 1, and 2) can be removed during the inference to save computational costs. Finally, 1×1×1 3D convolutions are added to the shortcut connections within each hourglass module (see the dashed lines in Fig 1) to improve performance without increasing computational costs. Since the 1×1×1 3D convolution only has 1/27 of the multiplication operations compared with 3×3×3 convolutions, it runs very fast, and the time can be neglected.

For the disparity regression, we use the soft argmin operation to estimate the disparity. The method is entirely differentiable and can obtain smooth disparity estimation results. First, the probability that each pixel belongs to each disparity value is calculated. After 3D CNN and upper sampling processing, each pixel’s matching cost volume under all disparities is obtained. The higher the costs are, the lower the probability of matching. Therefore, the negative value of the prediction costs is taken, and softmax is used. The operation is regularized to obtain the probability that each pixel belongs to a different disparity. Then, the probability value is used as the weight to sum the gap and obtain the disparity value at each pixel point, as shown in formula (1):

$$\hat{d}=\sum_{d=0}^{D_{max}}d\times \sigma \left(-C_{d}\right)$$ (1)

where $\hat{d}$ represents the predicted disparity value, *C*_*d*_ represents the matching costs under disparity *d*, and *σ*(·) represents the softmax operation. The mathematical expression of *σ*(·) is

$$\sigma \left(z_{j}\right)=\frac{e^{z_{j}}}{\sum_{k=1}^{K}e^{z_{k}}}\begin{array}{ccc} & & j=1,2,\ldots ,K \end{array}$$ (2)

For the loss function, the smooth L1 loss function is often used in the target detection boundary box regression problem. Compared with the L2 loss function, the smooth L1 loss function has better robustness and lower sensitivity to outliers. Due to the existence of the disparity regression, the smooth L1 loss function can be used for the model training in this paper, as shown in Formula (3):

$$L\left(d,\hat{d}\right)=\frac{1}{N}\sum_{i=1}^{N}smooth_{L_{1}}\left(d_{i}-{\hat{d}}_{i}\right)$$ (3)

Here,

$$smooth_{L_{1}}\left(x\right)=\{\begin{array}{c} 0.5x^{2},\begin{array}{cc} & \left|x\right|<1 \end{array}\\ \left|x\right|-0.5,\begin{array}{cc} & otherwise \end{array} \end{array}$$ (4)

where *N* represents the total number of pixels, *d* represents the ground truth disparity, and $\hat{d}$ represents the predicted disparity.

## Results

We validated our model on the stereo matching standard dataset using the PyTorch framework. This section shows the experiment and test results on the Scene Flow dataset and KITTI datasets. In Section 4.1 and section 4.2, the relevant information and implementation details of the two datasets are introduced. In Section 4.3, the influences of the parameters of ASPP on the network are explored. In section 4.4, the ablation experiment using the proposed network structure is conducted to test the impact of the network structure and parameter setting on the results. Section 4.5 shows the comparison of the results of the algorithm mentioned above in the KITTI rankings with other excellent algorithms.

### 4.1 Datasets and evaluation metrics

In this section, we evaluate the performance of our model on standard public datasets such as Scene Flow [11], KITTI 2012 [24], and KITTI 2015 [25].

#### 1) Scene flow dataset

All the scenes in the data set are generated by 3D animation software blender rendering. The data set is divided into three subsets: flying things 3D, driving, and monkaa. Furthermore, the data set contains 35454 training pictures and 4370 test pictures. With H = 540, W = 960 and dense and elaborate disparity maps provided as basic facts, the pixels’ disparity value is more significant than the limit set in this study, which will be ignored when calculating the loss. We removed some useless images according to the latest modifications of PSMNet made by the author. The numbers of images eventually used for training and testing was 34,881 and 4,248, respectively. The data set has the largest number of pictures, ensuring that the trained network model has a strong generalization ability and avoids overfitting.

#### 2) KITTI dataset

This data set is the most widely used computer vision algorithm evaluation data set in the world. The ground truth disparities in the data set are obtained by the following methods: Lidar scanning is used to obtain the three-dimensional coordinates of the spatial points, and then the corresponding disparity value is obtained through the reverse calculation of camera parameters. The KITTI 2012 data set contains 194 pairs of left and right images with the disparity truth value. Of these, 160 pairs of images are randomly selected as the model training set, and the remaining 34 pairs of images are used as the model verification set. The KITTI 2015 dataset contains approximately 200 pairs of images, and 160 pairs of images are randomly selected from the KITTI 2015 data set to train the model. The remaining 40 pairs of images are used for model verification. The pixel size of each pair of photos is 375 × 1241.

For the Scene Flow dataset, the evaluation metric used is usually the endpoint error (EPE). The EPE represents the average Euclidean distance between all pixels of the disparity estimation graph and the real disparity value. The EPE is calculated as follows:

$$EPE=\frac{1}{N}\sum_{i\in N}\sqrt{{\left(d_{i}-{\hat{d}}_{i}\right)}^{2}}$$ (5)

For KITTI 2012, the t-pixel error is used as the evaluation criterion. The t-pixel error represents the percentage of pixels with an EPE greater than t pixel units in the disparity estimation graph. Here, "all" represents all pixels participating in the error calculation, and "NOC" represents only the pixels of the nonoccluded area participating in the error calculation. For KITTI 2015, we use the percentage of incorrect pixels in the background (D1-bg), that in the foreground (D1-fg), or all the pixels (D1-all). If the disparity EPE is less than 3 px or less than 5%, the pixel is considered correct. The t-pixel error is calculated as follows:

$$tpx-Error=\frac{1}{N}\sum_{i\in N}\Phi \left(\left|d_{i}-{\hat{d}}_{i}\right|,t\right)\times 100\%$$ (6)

where

$$\Phi \left(p,q\right)=\{\begin{array}{c} 1,\begin{array}{cc} & p>q \end{array}\\ 0,\begin{array}{cc} & p\leq q \end{array} \end{array}$$

The total number of pixels *d*_*i*_ is the real disparity value of the *i*th pixel, and ${\hat{d}}_{i}$ is the predicted disparity value of the *i*th pixel.

### 4.2 Implementation details

Under the Windows 10 environment, the improved stereo matching network PSMNet proposed in this study is implemented using the Python deep learning architecture. The batch size is fixed to 4. In addition, we train all networks using two NVIDIA GTX 2080Ti GPUs with 2 training samples on each GPU, and the Adam optimizer is used. The optimization parameters are set to *β*_1_ = 0.9 and *β*_2_ = 0.999. During training, data enhancement operations are also conducted. Color, brightness, and contrast operations are transformed, and the pictures are randomly cut to 256 (H)×512 (W). These data enhancement operations help to develop a model that is more robust to light and noise. Similar to PSMNet [14], the maximum disparity of this paper is set to 192. Specifically, for the Scene Flow data set, training occurred for 10 epochs with a fixed learning rate of 0.001. The trained model can be directly used for testing. For KITTI 2015 and KITTI 2012, we fine-tune the network pretrained on the Scene Flow dataset for another 500 epochs, and a new network model is obtained. The learning rate is set to 0.001 in the first 200 epochs and 0.0001 in the remaining 300 epochs to prevent overfitting.

### 4.3 Experiment on the structure of ASPP

For the ASPP structure experiment, the expansion rate is a superparameter that needs to be determined manually. To achieve optimization, we test the expansion rate of the ASPP structure and set 6 groups of parameters. The first 3 groups of parameters are based on an expansion rate of 3, and the last 3 groups are based on an expansion rate of 2. The experimental results are shown in Table 2.

**Table 2:** The experiment of ASPP structure on Scene Flow.

dilation rate	EPE (Scene Flow)	KITTI 2015 (Val Err (%))
[3, 6, 9, 12]	1.07	1.95
[6, 12, 18, 24]	1.03	1.89
[9, 18, 27, 36]	1.05	1.97
[2, 4, 6, 8]	1.04	1.85
[4, 8, 12, 16]	0.99	1.78
[8, 16, 24, 32]	1.02	1.79

Table 2 shows that the expansion rate has a particular impact on disparity estimation accuracy. If the expansion rate is too low, the scope of the receptive field is small and not enough environmental information can be obtained; however, if the expansion rate is too large, the range of the receptive field is broad and it is easy to lose the detailed information of the target. Moreover, the parameter selection scheme based on an expansion rate of 2 is generally better than the parameter selection scheme based on an expansion rate of 3. Therefore, according to the experimental results, the range of the expansion rate is [4,8,12,16].

### 4.4 Ablation study

Table 3 gives the comparison results of different module combinations on the Scene Flow dataset. We use PSMNet, which is most similar to the network proposed in this paper, as a reference to evaluate the proposed system on the Scene Flow and KITTI2015 test sets.

**Table 3:** The results of the ablation comparison on Scene Flow.

Model	ResNeXt	ASPP	Our Stacked Hourglass	EPE (Scene Flow)	KITTI2015 (Val Err (%))	Params. (M)
Base (PSMNet)	×	×	×	1.09	1.98	5.2
Ours	√	×	×	1.07	1.97	4.6
	√	√	×	1.01	1.85	3.9
	√	√	√	0.99	1.78	3.7

As shown in Table 3, the first line uses the original PSMNet as a reference. In the second model, the ResNet module is replaced by ResNeXt, both of which have the same parameters, but the precision of ResNet is higher. In the third model, the SPP module is replaced by the ASPP module, and then a feature fusion module is used to fuse the feature information of different scales quickly and effectively. The last line is our improved PSMNet, which adds an auxiliary output module 0, removes the remaining connections between other output modules, and adds a 1×1×1 three-dimensional convolution to the quick links in each hourglass module. As shown in Table 3, our improved PSMNet delivers the best performance on Scene Flow.

In this subsection, the effectiveness of our design choice is empirically proven. According to the experimental results in Table 3, replacing ResNet with ResNeXt does not increase the parameters of the model and slightly improves the accuracy of the model. The introduction of the ASPP structure and feature fusion module can better extract regional contextual semantic information and effectively improve matching accuracy. In the stacked hourglass module, we remove the residual connection between different output modules, use a 1×1×1 3D convolution in each hourglass module, and add an auxiliary output module in the hourglass module (output module 0, see Fig 3) for the features of the pre-hourglass module. The extra additional loss makes the network learn better features at lower layers, which also benefits the final prediction. Furthermore, the extra additional loss also makes the accuracy of the system designed in this study exceed that of the reference network PSMNet. As listed in the fifth row of Table 3, the 3 px error rate on the KITTI 2015 validation set decreases from 1.98 to 1.78, and the end point error on the Scene Flow test set decreases from 1.09 to 0.99.

### 4.5 Performance comparison with the state-of-the-art

**Fig 4:**
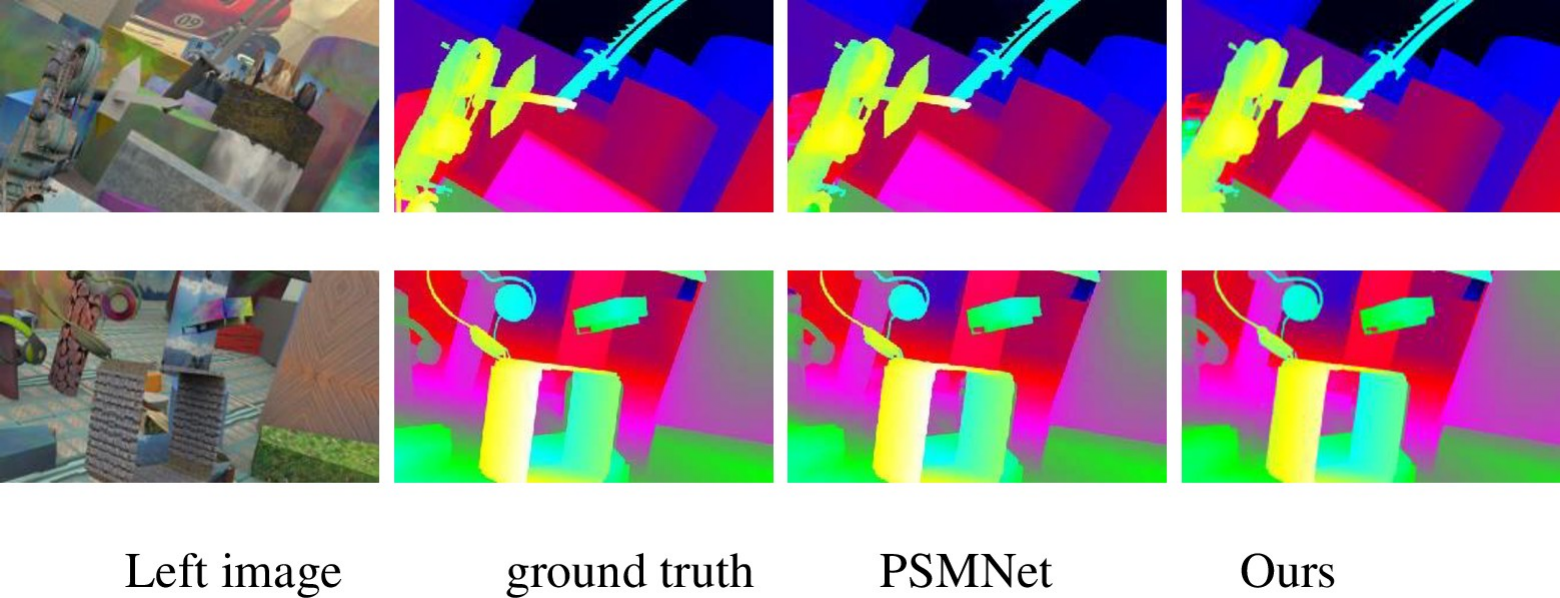
Qualitative evaluation results on Scene Flow. The first column shows the left images, the second column shows the ground truths, the third column shows the disparity maps estimated using PSMNet, and the fourth column shows the results of our model.

**Fig 5:**
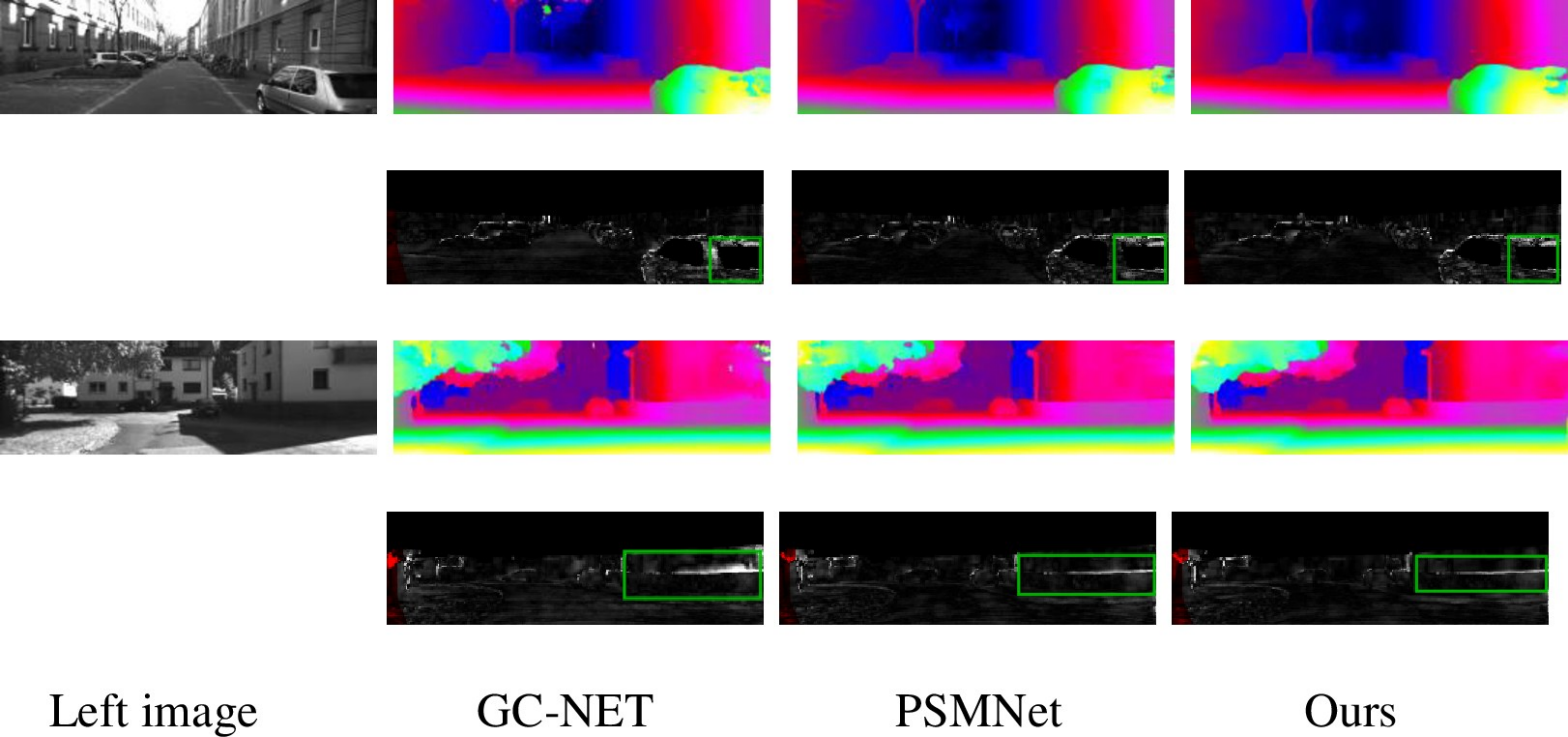
Qualitative evaluation results on the KITTI 2012 dataset. The first column shows the left images. For each photo, the first row shows the pseudocolor images for predicting the disparity map, and the second row shows the error maps. From left to right are the results of GC-NET [18], PSMNet [13], and our method, respectively.

Table 4 shows that compared with the current end-to-end algorithm, the matching accuracy of the proposed algorithm can still reach the top level under the condition of a minimum number of parameters. Compared with PSM, the number of parameters of this algorithm is reduced by 25%; and the endpoint error is 0.99, which is reduced 0.1 and far lower than the end-to-end error of GC-Net [18]. Table 4 shows that the number of parameters of the algorithm is not directly proportional to the matching accuracy. For example, CRL [12] does not consider the physical meaning of disparity itself and simply stacks many convolution layers to estimate the disparity value, which makes the model cumbersome and inefficient. Thanks to the practical design of the feature extraction network and similarity calculation algorithm, this paper’s algorithm can obtain a more accurate disparity map when the number of parameters is only one-twentieth of that of CRL. This fully reflects the efficiency and accuracy of the proposed method. Fig 4 illustrates some examples of the disparity maps estimated by the proposed PSMNet and ours. Our method obtains more robust results than PSMNet, as indicated by the green boxes in Fig 5.

**Table 4:** Comparison of the EPEs of different networks on the Scene Flow dataset.

	PSMNet [13]	GC-Net [18]	CRL [12]	Our Model
Params. (M)	5.2	3.5	75	3.9
EPE	1.09	2.51	1.67	0.99

## Discussion

**Fig 6:**
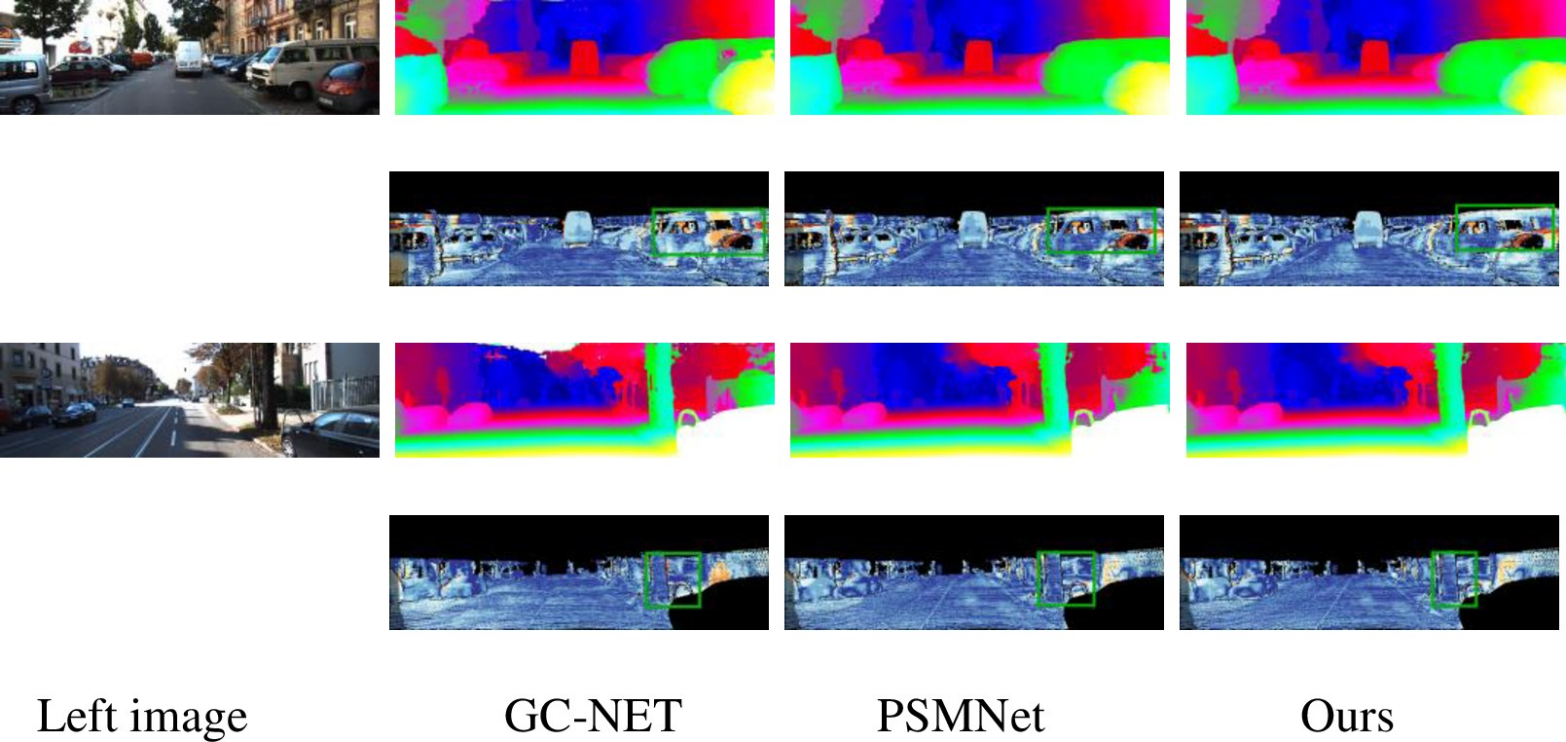
Qualitative evaluation results on the KITTI 2015 dataset. The first column shows the left images. For each photo, the first row shows the pseudocolor images for predicting the disparity map, and the second row shows the error maps. From left to right are the results of GC-NET [18], PSMNet [13], and our method, respectively.

Intuitively, this algorithm can obtain a dense disparity map, preserve details such as the tree trunk and window, and have a good matching effect. The disparity map obtained by this algorithm is smoother than that obtained by PSMNet. For example, the images in Fig 5 show a more evident and denser car contour. Compared with the PSMNet method, the matching accuracy of this algorithm is slightly improved. Due to the addition of the ASPP structure and feature fusion module, the matching accuracy in some ill-conditioned regions is improved. There are black walls and a large area shadow in the third line of Fig 5. There are almost no apparent texture features to assist in finding the corresponding matching points. It is easy to confuse the black wall with the shadow. The reflective glass and fence areas in Fig 6 are also ill-conditioned areas. However, the network proposed in this paper obtains a better disparity map. Generally, this algorithm’s matching effect is improved compared with those of other methods, and matching accuracy is improved.

For the KITTI2012 and KITTI2015 datasets, the migration learning method is adopted. The Scene Flow training model is fine-tuned on the KITTI 2012 and KITTI 2015 training sets for 300 cycles, and a new network model is obtained. We apply the trained model to the test set, obtain the test results, upload them to the KITTI website for quantitative evaluation and compare the results with those other excellent algorithms, including CRL [12], GC-NET [18], DispNetC [11], and PSMNet [13]. Tables 5 and 6 show some algorithms’ performance on the KITTI 2012 and KITTI 2015 datasets. As Table 5 shows, the overall three-pixel error of our method is 1.76%. Under the same scale parameters, our method achieves the best performance and runs faster than most other methods. Compared with the previous network, the proposed system obtains a larger improvement in precision, and the number of parameters is the lowest. As Table 5 shows, compared with the reference network PSMNet, the overall mismatch ratio is reduced from 2.32% to 2.25%. In terms of running time, to ensure the reliability of the data, the running time of PSMNet in Table 6 is 0.41 s under the same test conditions, and the calculation time of the algorithm proposed in this study is 0.35 s. Efficiency is improved by approximately 15%. Compared with GC-NET, the disparity map’s mismatch rate is reduced by 0.62%, and the running speed is increased nearly three times.

**Table 5:** KITTI 2012 quantitative assessment results.

Method	>2px		>3px		>5px		Mean Error		Runtime (s)
	Noc	All	Noc	All	Noc	All	Noc	All	
PSMNet [13]	2.44	3.01	1.49	1.89	0.90	1.15	0.5	0.6	0.41
GC-Net [18]	2.71	3.46	1.77	2.30	1.12	1.46	0.6	0.7	0.9
SeStereo [26]	2.66	3.19	1.68	2.03	1.00	1.21	0.5	0.6	0.6
DispNetC [11]	7.38	8.11	4.11	4.65	2.05	2.39	0.9	1.0	0.06
Our Model	2.43	3.05	1.42	1.76	0.83	1.16	0.5	0.5	0.35

**Table 6:** KITTI 2015 quantitative assessment results.

Method	All (%)			Noc (%)			Runtime (s)
	D1-bg	D1-fg	D1-all	D1-bg	D1-fg	D1-all	
PSMNet [13]	1.86	4.62	2.32	1.71	4.31	2.14	0.41
GC-Net [18]	2.21	6.16	2.87	2.02	5.58	2.61	0.9
CRL [12]	2.48	3.59	2.67	2.32	3.12	2.45	0.47
DispNetC [11]	4.32	4.41	4.34	4.11	3.72	4.05	0.06
Our Model	1.81	4.39	2.25	1.68	4.17	2.08	0.35

## Conclusions

Aiming at the problems of a high number of parameters and insufficient accuracy in the stereo matching networks, this paper proposes an efficient and accurate stereo matching algorithm based on an improved PSMNet. The feature extraction module uses ResNeXt instead of ResNet. With the same parameters, ResNeXt achieves higher precision. By using ASPP and the feature fusion module, the network’s receptive field is effectively expanded, and the feature information of different scales is extracted to construct a four-dimensional matching cost volume. We also improve the stacked hourglass network, further enhance performance and reduce reasoning time. The performance of the proposed method on the KITTI 2012, KITTI 2015, and Scene Flow data sets is verified and compared with the performances of several types of typical deep learning-based methods. The proposed algorithm has achieved the optimal performance in overall accuracy, especially compared with the reference methods; effectively improved the detail areas and challenging weak texture plane areas, such as the disparity precision area; and reduced the running time. Furthermore, with the extensive application of unsupervised networks, stereo matching can gradually no longer rely on large-scale data sets with real values, making network training easier and improving the system’s generalization ability. This will be the target of our next research.
